# Editorial

**Published:** 1864-10

**Authors:** 


### Editorial.

### WHY ARE NOT OUR DENTAL SCHOOLS BETTER SUSTAINED BY THE PROFESSION?

In attempting a reply to this question, we can not expect in a few brief sentences to present everything that would be relevant to the subject, but intend here only to present a few of the more prominent points of the subject.

And, first, this want of support does not occur because the advantages derivable from dental colleges are not needed, for almost every one in the profession will admit his own deficiencies and short comings in attainments, and, too, in many of the branches fully dwelt upon in the schools. If this is true, in reference to those already in the profession, who have been practicing for years, how much more is it true of those who enter the profession with only a few superficial ideas in regard to practice, which have been obtained through a brief tutorage of some one man who is not overstocked with the erudition or science of our profession.

As a rule, the best men in our profession will not take a student with a view of completing his professional education.

The fact that any dentist will take a student, with the promise to thoroughly prepare him for the practice of his profession, in all that pertains to it, gives the most indubitable evidence that his own conceptions of the proper attainments, are very meagre indeed.

In our opinion there is no man on earth fully competent to properly teach, or even approximate it, all the branches connected with our profession; we do not know of a man fully competent to teach half of them, even if he should devote his whole time to it.

The young man then, seeking to enter our profession, can no where secure the attainments he ought to possess outside of a well regulated college or academy. One reason then why our dental schools are not better sustained is that so many in the profession fail to comprehend the attainments essential to constitute a dentist; they so generally entertain the idea, which is far too prevalent everywhere, that to become a dentist is a small thing. No man, who fully comprehends the subject, will dare set himself up, for a whole teacher. We need hardly say here that the student's idea is at fault, for the impress of his first teacher is usually so deeply stamped upon him, that it is never removed.

The objection is sometimes made that our colleges are not as efficient as they should be; that we grant at once, but then, the course of instruction, given even in the poorest of them, is infinitely better than it is possible for any one man to give in a private office.

But why are our colleges inefficient? In answer to this: How are they sustained by the profession at large? Do the members of the profession, as a general thing, endeavor to fill up the classes, and in that way, and by their sympathy, encouragement, and their money if need be, endeavor to build up our schools, and thus bring them to the highest point of efficiency, and thus make them monuments of professional energy, and institutions for good, of which every member should be proud.

We seem to have forgotten, that as one member progresses, all progress, and that while we are making efforts to elevate others, we ourselves are becoming elevated.

Our schools, then, are not sustained; because as a profession we do not appreciate the advantages derivable from them. This feeling toward our schools in the profession is very difficult to account for, except it may be from ignorance, narrow-minded prejudice and jealousy.

There is every inducement, from the highest to the lowest, for men entering our profession, or those already in it, to make the highest possible attainments.

Every one, of course, understands that the more extended one's attainments, the more power he has—the more he can accomplish. Such an one possesses a confidence, and a self-reliance, which others can not have.

It is true the ignorant man will be many a time self confident, but it is always based upon pride, conceit and ignorance.

Does a man wish to occupy a good position in his profession,—and who does not—he must make that position by attainments.

And who does not wish to occupy an honorable position with other professions; the dentist can only do so, by the possession and proper use, of professional and collateral attainments; and ere long, and in many places even now, the ignorant pretender is not able to palm off his ignorance and base deceptions for that which is true and valuable, upon the people; the people are becoming educated, and then woe to pseudo dentistry and pseudo dentists.

Does a man desire to make money; the greater the attainments the greater the power to do that. Why do so many in our profession go almost beggarly through life? because with few exceptions they deserve nothing better, they are content with the crumbs of professional attainments,—while whole loaves lie within their reach,—and of course the public will give them the beggar's portion.

There may be of course to this a few exceptions; misfortune sometimes comes with a rush, beneath which any one must bow. Some again, in our profession as elsewhere, will almost spend their lives and their all for the good of others.

No one who has ability and can make his services valuable to his fellow beings, will fail to be appreciated, or sustained in every sense. It is sometimes said of men in our profession, of limited attainments, that they make money; that is true of men in every avocation in life; when men have not power and ability, they may employ strategy and trickery to accomplish that which ought to be otherwise done. No honest man feels well, in the enjoyment of that for which he has not rendered an equivalent.

Now in regard to our schools, they are not sustained by our profession as they should be, nor are they efficient as they would be, were they properly supported. Nor do those in the profession derive a tithe, the benefit they might were they disposed to have it otherwise. We must say there are some noble exceptions in the profession, and but for these, we could have no dental colleges. T.

### THE DENTISTS' MEMORANDUM AND ENGAGEMENT BOOK.

The Dentists' memorandum and engagement book, for 1865 will be issued in a few days and ready for distribution.

It is printed from the same plates as the edition published in 1863, in this city, by Dr. Cleveland. Those familiar with that, need no description of this edition, as it will be the same throughout. It contains a large amount of valuable matter, in the form of directions, recipes, formulas, &c., all of which is valuable to the dentist for ready reference. It is printed on the best quality of paper and neatly bound. No dentist in full and regular practice can do without such a work. It will be for sale at the dental depots ; we can also furnish to any who may desire. Price $1.50.

T.

### THE DENTAL LUMINARY.

The second number of this work for popular reading has just been issued, by the "Cincinnati Dental Society." Its contents are such as will prove valuable to any reader who desires to take care of and preserve his teeth. Some systematic method of imparting information to the people upon this subject should be adopted by every dentist, and there is none perhaps better than distributing such works as the above. This work is stereotyped, contains twenty-four pages, and is of convenient form, smaller page than the first number, and is neatly gotten up. It can be furnished to any member of the profession, with their card on the cover for $5.50 per hundred copies, and if paper should come down, at a less price. We shall be pleased to fill any orders that may be given for it. The committee having the matter in charge consist of Drs. A. Berry, M. Rogers and J. Taft.

